# Nanostructure, Self-Assembly, Mechanical Properties, and Antioxidant Activity of a Lupin-Derived Peptide Hydrogel

**DOI:** 10.3390/biomedicines9030294

**Published:** 2021-03-13

**Authors:** Raffaele Pugliese, Anna Arnoldi, Carmen Lammi

**Affiliations:** 1 NeMO Lab, ASST Grande Ospedale Metropolitano Niguarda, 20162 Milan, Italy; 2 Department of Pharmaceutical Sciences, University of Milan, 20133 Milan, Italy; anna.arnoldi@unimi.it

**Keywords:** self-assembling peptides, supramolecular hydrogels, nano-nutraceuticals, rheology, lupin peptides, antioxidant activity, bioactivity, mechanical properties

## Abstract

Naturally occurring food peptides are frequently used in the life sciences due to their beneficial effects through their impact on specific biochemical pathways. Furthermore, they are often leveraged for applications in areas as diverse as bioengineering, medicine, agriculture, and even fashion. However, progress toward understanding their self-assembling properties as functional materials are often hindered by their long aromatic and charged residue-enriched sequences encrypted in the parent protein sequence. In this study, we elucidate the nanostructure and the hierarchical self-assembly propensity of a lupin-derived peptide which belongs to the α-conglutin (11S globulin, legumin-like protein), with a straightforward N-terminal biotinylated oligoglycine tag-based methodology for controlling the nanostructures, biomechanics, and biological features. Extensive characterization was performed via Circular Dichroism (CD) spectroscopy, Fourier Transform Infrared spectroscopy (FT-IR), rheological measurements, and Atomic Force Microscopy (AFM) analyses. By using the biotin tag, we obtained a thixotropic lupin-derived peptide hydrogel (named BT13) with tunable mechanical properties (from 2 to 11 kPa), without impairing its spontaneous formation of β-sheet secondary structures. Lastly, we demonstrated that this hydrogel has antioxidant activity. Altogether, our findings address multiple challenges associated with the development of naturally occurring food peptide-based hydrogels, offering a new tool to both fine tune the mechanical properties and tailor the antioxidant activities, providing new research directions across food chemistry, biochemistry, and bioengineering.

## 1. Introduction

Food proteins not only supply nutrients, but also provide numerous beneficial effects through their impact on specific biochemical pathways. Most of these activities are due to peptides encrypted in the parent protein sequences, which are delivered by digestion, absorbed intact by intestinal cells, and transported to their target organs where they exert their biological activity [1,2]. Over the years, numerous bioactive peptides have been identified in protein hydrolysates from various foods [3].

In this context, lupin is a grain legume belonging to the *Fabaceae* family, whose food applications have improved during the last two decades owing to the protein content ranging from 35% to 40%, and the lack of commercially available genetically modified varieties. Many in vitro, in vivo, and human studies have highlighted the health benefits of lupin protein consumption [4,5,6,7]. Notably, the hydrolysis of lupin proteins using trypsin generated a mixture composed of about 3000 different peptides, which were identified by peptidomic analysis [4]. Biochemical and cellular investigations revealed its hypocholesterolemic and hypoglycemic activities [4,8,9]. Transepithelial transport experiments performed using a differentiated model of Caco-2 cells indicated that only eleven tryptic peptides are able to cross the intestinal cells [10].

Among the absorbed peptides, the LNALEPDNTVQSEAGTIETWNPK (named T13) stands out for its interesting structural properties [11]. Indeed, T13, which is composed of 23 amino acids, belongs to the α-conglutin (11S globulin, legumin-like protein), which is a lupin globulin exerting the function of a seed storage protein (Figure S1). It shows a pI equal to 3.58, a net charge of −3, and a theoretical hydrophobicity equal to +26.02 kcal * mol^−1^. In addition, its computational model was created by homology modeling techniques and the high percentage of sequence homology found with the employed template, the soybean proglycinin (PDB code 1UCX), made it possible to achieve a model of reasonable quality, additionally supported by molecular dynamic (MD) simulations, suggesting that T13 was shaped as a β-hairpin [11].

Given its peculiarity of spontaneously organizing into ordered β-hairpin structures, we thought it was a promising candidate capable of creating nanostructures suitable for the life sciences [12], for metabolic diseases [13], for cell culture [14], or as a nanomoisturizer for wound healing [15] or skin care [16]. However, despite the β-hairpin secondary structures that may be involved in the self-assembly phenomenon of protein-based materials, the practical application of this T13 peptide is limited due to its intrinsic instability in solution, low performance, and low biomechanical features.

Since biotin—composed of ureido and tetrahydrothiophene rings sideways fused and by a valeryl chain—proved to facilitate and stabilize self-assembling peptide nanofibers (SAPs) [17,18,19] without altering their biocompatibility, we added to the T13 peptide an N-terminal biotinylated oligoglycine tag to foster its self-assembly propensity, and to enhance its biomechanical properties (Figure S2).

It is well established that the chemical structure of biotin possesses the ability to form H-bonds and hydrophobic contacts through the ureido ring and the tetrahydrothiophene ring, respectively. Indeed, by using Molecular Dynamics (MD) simulations it was demonstrated that the concomitance of such interactions plays a key role in the formation of dense H-bond networks and of a “hydrophobic wall” synergically triggering a disorder-to-order transition in SAP molecules containing N-terminal biotin cap [20]. Saracino and Gelain empirically demonstrated that the presence of a biotin N-terminus tag on bone marrow homing peptide (BMHP-1) evolving towards structured β-sheets against the globular aggregates, was formed by the same unbiotinylated peptides [20]. Furthermore, as experimentally observed, the authors pointed out that biotin significantly supports the hierarchical self-assembly into ordered β-sheet structures and improves the biomechanics of the final scaffolds [17,18]. Jekhmane et al. also reported that by using solid state NMR, that biotinylated scaffolds assembly degree, mechanics, and homogeneity correlate with favorable neural stem cell behavior [19]. In another effort, Davis et al. designed biotinylated peptide nanofibers for the prolonged delivery of insulin-like growth factor 1 (IGF-1) for improving cell therapy for myocardial infarction [21].

Herein, by using the advantage of the biotin tag, we obtained a thixotropic lupin-derived peptide hydrogel (named BT13) with tunable mechanical properties. The secondary structures, biomechanics, and nanostructure morphologies of T13 and BT13 hydrogel were extensively characterized by Fourier transform infrared spectroscopy (FT-IR), circular dichroism (CD), oscillatory stress rheology, and atomic force microscopy (AFM). Finally, we demonstrated for the first time the antioxidant activity of a food-derived peptide hydrogel. To achieve this goal, the 2,2-diphenyl-1-picryl-hydrazyl-hydrate (DPPH) assay was used. The DPPH radical scavenging assay is one of the most commonly used antioxidant procedures based on single electron transfer, due to its ease of performance, rapidity, automation potential, reproducibility, and usability at ambient temperatures [22,23].

This study may facilitate the development of naturally occurring peptide-based materials with controlled and tunable biomechanical and biological properties, and broaden the number of applications of self-assembling peptide hydrogels and nano-nutraceuticals.

## 2. Materials and Methods

### 2.1. Materials

All reagents and solvents used for the peptide characterizations and in vitro experiments were purchased from commercial sources and used without further purification.

### 2.2. Peptide Preparation

The peptides were purchased from GenScript Biotech Corporation (Piscataway, NJ, USA). The purity of lyophilized peptides (>95%) was tested by binary HPLC and by Agilent 6520 LCMS mass spectrometry (Figure S3). Lyophilized peptides were dissolved at 0.5%, 1%, 3%, and 5% (w/v) in distilled water (GIBCO^®^ Thermo Fisher Scientific, Waltham, MA, USA), and then stored at +4 °C overnight before use. For hydrogel preparation, 1X Dulbecco’s Phosphate Buffered Saline (DPBS) (Ca^2+^/Mg^2+^-free) was added dropwise to the peptide sample (100 μL), and vigorously stirred for 5 min.

### 2.3. Thioflavin T (ThT) Spectroscopy Assay

ThT analysis of peptides was performed to assess the presence of amyloidogenic fibril structures. Peptide samples (40 μM) were mixed with the ThT solution (20 μM) and stirred for 4 min, as previously reported [24]. ThT fluorescence intensity was recorded using a Synergy plate reader (Biotek, Bad, Friedrichshall, Germany) with λ_ex_ = 440 nm (5 nm bandpass) and λ_em_ = 482 nm (10 nm bandpass), over 60 s at 25 °C. Measurements were normalized over ThT-alone fluorescence and processed with OriginLab™ 8 software.

### 2.4. Fourier Transform Infrared Spectroscopy (FT-IR)

The FT-IR spectra of peptides were obtained in attenuated total reflection (ATR) using a PerkinElmer Spectrum 100 spectrometer. Twenty acquisitions were recorded for each spectrum, using the following conditions: 4 cm^−1^ spectrum resolution, 25 kHz scan speed, 1000-scan coaddition, and triangular apodization. All of the obtained spectra were reported after ATR correction, smoothing, and automatic baseline correction using Origin™ 8 software.

### 2.5. Circular Dichroism Spectroscopy (CD)

The CD spectra of peptides were recorded on a Jasco J-815 (Jasco Corp., Tokyo, Japan) spectropolarimeter using a 0.1 mm quartz cuvette, as previously reported [25]. Spectra were collected in the 180–300 nm spectral range and averaged over three scans at room temperature. All the scans were carried out at a scan speed of 50 nm/min, with a bandwidth of 1 nm and time-response parameters set to 2 s. A reference spectrum of distilled water was recorded and subtracted from each spectrum. The estimation of the peptide secondary structure was achieved by using a chemometrics method (http://bestsel.elte.hu/index.php). All of the obtained spectra were reported using Origin™ 8 software.

### 2.6. Mechanical Testing

Rheological properties of peptides were carried out using a stress/rate-controlled Kinexus DSR Rheometer (Netzsch, Selb, Germany) equipped with a parallel plate geometry (acrylic diameter 20 mm; gap 34 μm). All measurements were obtained at 25 °C using a Peltier cell in the lower plate of the instrument to control the temperature during each test. Strain sweep experiments were performed on samples from 0.1% to 1000% strain to determine the limit of the linear viscoelastic regime (LVR). To evaluate the storage (G’) and loss (G’’) moduli increase, frequency sweep experiments were recorded as a function of angular frequency (0.1–100 Hz) at a fixed strain of 1%. To test the thixotropy of peptides, shear-thinning tests were performed by a series of peak hold tests in which shear rates were kept constant, as previously reported [26]. Briefly, firstly a shear rate of 0.01 s^−1^ was applied for 60 s, and then a shear rate of 5.3 s^−1^ was applied for 20 s, in order to simulate the shear rate inside a syringe barrel. Subsequently, a high shear rate of 1000 s^−1^ was applied for 20 s to simulate the purge injection of the solution. Afterwards, a shear rate of 5.3 s^−1^ (20 s) was applied again, thus mimicking the flow of peptide solution out of the needle. Lastly, a shear rate of 0.01 s^−1^ was used to simulate the low shear condition of the solution during injection. Each experiment was done in triplicate, and data were processed using Origin™ 8 software.

### 2.7. Atomic Force Microscopy (AFM)

AFM measurements were captured in tapping mode using a Tosca system (Anton Paar, Graz, Austria) using single-beam silicon cantilever probes. AFM images were taken by depositing 5 μL solutions (final concentration of 0.01% w/v) onto freshly cleaved mica. The samples were kept on the mica for 5 min; subsequently they were rinsed with distilled water to remove loosely bound peptides, and then dried under ambient conditions for 24 h. The morphological parameter analysis of the AFM data was performed using the Matlab-based open-source software FiberApp.

### 2.8. 2,2-Diphenyl-1-picrylhydrazyl (DPPH) Assay

The DPPH assay to determine the antioxidant activity was performed by a standard method with some slight modifications [27]. BT13 (0.5%, 1%, and 3% w/v) and RADA16 (1% w/v) hydrogels were deposited in a 96-well half area plate, respectively. The reaction for scavenging DPPH radicals was performed adding 40 µL of DPPH solution in the dark at room temperature, and the absorbance was measured at 520 nm after 30 min incubation using the Synergy H1 plate reader.

### 2.9. Statistical Analysis

Statistical analyses were carried out by one-way ANOVA followed by Tukey’s post-hoc test (GraphPad Prism 8). Values were expressed as means ± SD; *p*-values < 0.05 were considered to be significant.

## 3. Results and Discussion

### 3.1. Supramolecular Organization of Lupin-Derived Hydrogel

To gain insights into the supramolecular and global arrangement of the BT13 peptide following the biotin tag, we used circular dichroism (CD), FT-IR spectroscopy, thioflavin-T (ThT)-binding assay, and atomic force microscopy (AFM) morphological analysis.

**Figure 1:**
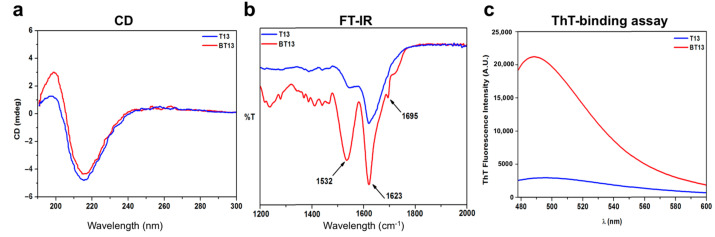
Supramolecular organization of T13 and BT13 peptide solutions. (**a**) Circular dichroism (CD) spectra of T13 (in blue) and BT13 (in red) suggesting the presence of β-sheet secondary structures. (**b**) FT-IR spectra of T13 and BT13 peptides with a characteristic β-hairpin conformation for T13, and typical anti-parallel β-sheet conformation for BT13 peptide. (**c**) ThT-binding assay of T13 and BT13 peptides, showing a high amyloid-binding emission signal (centered at 490 nm) of BT13 compared to T13 peptide.

To explore the effect of the biotin tag on the secondary structure conformation of BT13 assemblies, CD spectra were recorded in the far UV region of 180–300 nm (Figure 1a). As expected, for T13 peptide (in blue) a positive and negative Cotton effect was observed at 198 nm and 215 nm, respectively, characteristic of β-sheet signals. The CD spectra for BT13 peptide (in red) showed one minimum peak at 215 nm and one more pronounced maximum peak at 195 nm, thus indicating highly ordered β-sheet-rich assemblies [28]. In order to further explore the structural arrangement of both peptides in solution, we carried out FT-IR spectroscopy tests (Figure 1b). The T13 peptide showed one broad peak at 1623 cm^−1^, and a lower stretching peak at 1532 cm^−1^, characteristic of the β-hairpin conformation [29]. On the contrary, FT-IR spectra of BT13 peptide exhibited a sharp amide I band at 1623 cm^−1^ with a shoulder at 1695 cm^−1^, indicating predominantly antiparallel β-sheet features, typical of SAP-based hydrogels [30,31]. The band at 1532 cm^−1^ in the amide II region also confirmed the β-sheet aggregation of BT13 peptide, in agreement with the CD spectra analysis. These changes in the FT-IR absorption peaks may reflect the effect of biotinylated oligoglycine tag on the BT13 assemblies that stabilize the β-sheet secondary structures of BT13, thus fostering its self-assembly propensity.

Since the β-sheet structure is characteristic of amyloid-like fibers, which are well established to be one of the basic functional motifs of self-assembling peptide molecules [32], we examined this behavior on BT13 peptide using the thioflavin-T (ThT) binding assay, an amyloid-specific fluorescent dye [33,34]. Staining the BT13 peptide sample with ThT resulted in high fluorescence levels compared to T13 peptide (Figure 1c), with a typical amyloid-binding emission signal (centered at 490 nm), establishing its β-sheet-rich amyloid-like nature, similar to other self-assembling peptides found in the literature [35,36,37,38,39,40].

**Figure 2:**
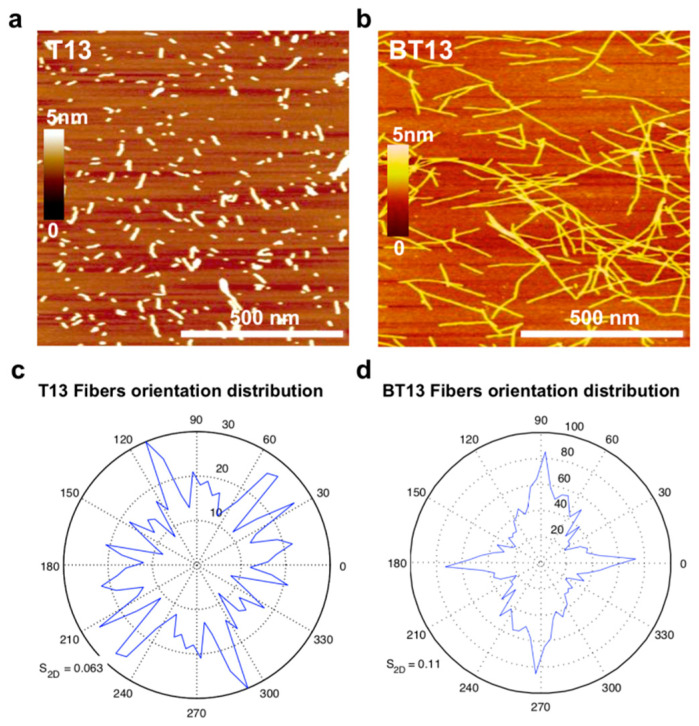
Morphological organization of T13 and BT13 peptide nanostructures. Atomic force microscopy images of (**a**) T13 and (**b**) BT13 peptides. Orientation distribution of (**c**) T13 and (**d**) BT13 peptides showing that the N-terminal biotin tag triggers a disorder-to-order transition in the BT13 assemblies. Scale bar, 500 nm.

Finally, we sought to investigate the nanostructures of T13 and BT13 peptides by AFM, which allows for examination of the fiber distribution of the assemblies (Figure 2a,b). T13 peptide yielded short nanorods with 1.4 ± 0.5 nm height. In contrast, BT13 led to the formation of a longer and clustered-bundle network of nanofibers compared to T13 peptide solutions. However, morphometric analysis showed that the height of BT13 fibers remained almost unchanged (2.5 ± 0.33 nm). It is interesting to observe that the BT13 fibers show a more oriented distribution compared to the T13 peptide (Figure 2c,d), which is probably due to the presence of an N-terminal biotin tag, which (1) favors the assembly, and (2) triggers a disorder-to-order transition among the peptide bundles, as previously reported [41].

### 3.2. Mechanical Properties of Lupin-Derived Hydrogel

Rheological studies were conducted to understand the role of biotin in the mechanical properties of BT13 hydrogel.

Rheological measurements of the storage (G’) and loss (G’’) moduli are commonly used to characterize viscoelastic and mechanical properties of hydrogels [42]. Here, G’ reflects the stiffness, while G’’ represents the energy dissipated during the oscillatory test and correlates with the liquid-like response of the hydrogel. The ratio between G’ and G’’ (i.e., tanδ) provides insights into the viscoelastic profile, that is, whether a material behaves as an elastic solid (G’ > G’’, tanδ < 1) or as a viscous liquid (G’ < G’’, tanδ > 1). Usually, in β-sheet-rich SAPs after self assembly, interactions among assembled fibers lead to the formation of an entangled nanofibrous network and increased values of G’ [37].

**Figure 3:**
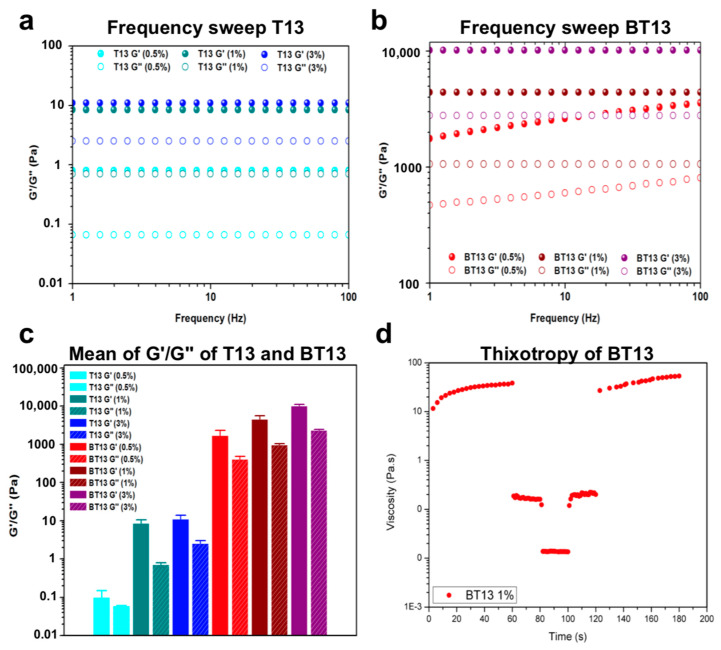
Rheological studies to evaluate the mechanical properties of BT13 hydrogels. Frequency-dependent oscillatory rheology (0.1–100 Hz) of (**a**) T13 and (**b**) BT13 peptides at 0.5%, 1%, and 3% (w/v). (**c**) Average values of storage (G’) and loss moduli (G’’) of T13 and BT13 peptides at 0.5%, 1%, and 3% (w/v). (**d**) Thixotropy test of BT13 hydrogel.

First, we conducted strain sweep experiments to investigate the linear viscoelastic regime (LVR) of both peptides (Figure S4). Next, we investigated G’ and G’’ moduli as a function of angular frequency (1–100 Hz, fixed strain 1% within the LVR) at different concentrations (0.5%, 1%, and 3% w/v) of the T13 and BT13 peptides (Figure 3). The frequency sweep data of both peptides suggested that G’ and G’’ are independent of applied frequency, showing a predominantly elastic-like behavior (G’, full dots) relative to the viscous component (G’’, empty dots). However, T13 peptide, due to its intrinsic instability in solution, showed low biomechanical features with G’ values ranging from 1 to 10 Pa, implying a weaker tendency to assemble (Figure 3a), as previously reported in AFM analysis. In contrast, the assemblies formed by BT13 peptide exhibited increased mechanical properties as a function of peptide concentration with G’ values of 2 kPa (for 0.5%), 4.5 kPa (for 1%), and 11 kPa (for 3%) (Figure 3b), thus indicating the formation of stiffer assembled gels compared to the T13 peptide (Figure 3c).

This notable increase in the storage modulus of BT13 is likely due to the presence of biotin, which fosters self-assembly propensity, increasing the noncovalent interactions among β-sheet-rich peptide bundles, thus leading to the formation of an entangled and stable nanofibrous network, in accordance with the AFM analysis.

It should be noted that above 5% (w/v), the BT13 peptide becomes too viscous and is no longer soluble in water, which is probably due to its high molecular weight (2753.01 g*mol^−1^). Despite the critical gelling concentration (CGC) of BT13 at 5% (w/v), this peptide’s presence improved mechanical properties at low concentrations, which is desirable for biomedical applications in addition to being more cost effective and theoretically more cytocompatible.

As a next step, since injectable hydrogels have gained increasing amounts of attention in the fields of biomedicine and bioprinting, and in the delivery of drugs, cells, biomolecules, and growth factors due to their minimally invasive delivery method [43], in order to evaluate the propensity of BT13-based hydrogel to recover its initial viscosity after injection, we performed thixotropy tests. In this test, the injection conditions were simulated through a series of constant shear rate tests (see Materials and Methods for further details). We observed that the viscosity of BT13 decreased and nearly returned to original values respectively during and after injection. This rapid viscosity recovery hints at the capability of BT13 to retain the three-dimensional network after gel−sol−gel conversion, particular to thixotropic SAP-based hydrogels.

### 3.3. Intrinsic Antioxidant Property of Lupin-Derived Hydrogel

**Figure 4:**
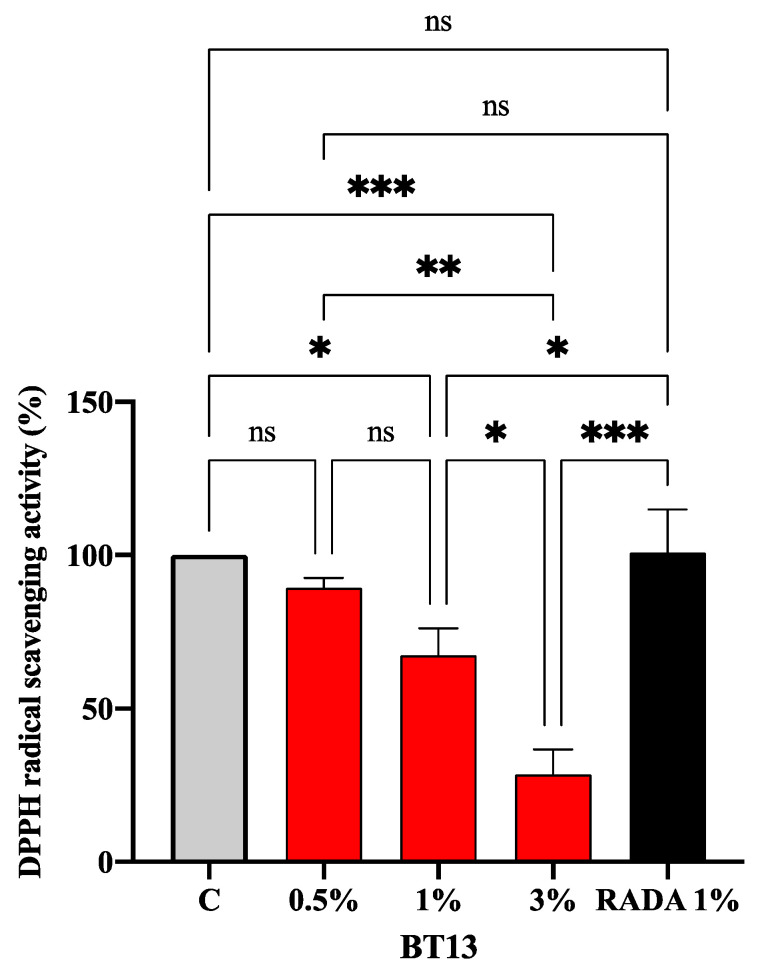
In vitro antioxidant power evaluation of BT13 by DPPH assay. The data points represent the means ± SD of four independent experiments in triplicate. Data were statistically analyzed by one-way ANOVA followed by Tukey’s post-hoc test. (*) *p* < 0.5; (**) *p* < 0.1; (***) *p* < 0.001. ns: not significant; C: control sample.

In order to assess the intrinsic antioxidant property of BT13 hydrogel (0.5%, 1%, and 3% w/v), DPPH assay was used. Experiments were carried out using RADA16 (1% w/v) as a reference, since it is a well-known and widely characterized SAP-based hydrogel [44]. Results clearly highlighted that BT13 displays an intrinsic antioxidant activity, which increases according to the concentration. On the contrary, RADA16 is completely ineffective. In detail, BT13 scavenged the DPPH radicals by 10.6% ± 3.3%, 32.6% ± 8.7% (*p* < 0.5), and 71.5% ± 8.2% (*p* < 0.001) at 0.5%, 1%, and 3% (w/v), respectively (Figure 4). Statistical analysis indicated that no significant difference was observed between 0.5% and 1% of BT13, whereas a significant difference was observed when BT13 was tested at 3%.

A preliminary screening of the T13 structure using BIOPEP (www.uwm.edu.pI/biochemia) suggested that it might be a bioactive peptide (Table 1). Indeed, T13 in plain solution reduced the radical DPPH by 29.2% ± 1.4% and 34.6% ± 3.2% at 0.5% and 1%, respectively (Figure S5), confirming the BIOPEP screening. In addition, this result highlights the fact that the antioxidant property of BT13 is intrinsic to its peptide sequence. In fact, within its sequence some bioactive motifs have already been reported as antioxidant and inhibitors of ACE, alpha-glucosidase, and DPP-IV enzymes, respectively. Notably, as reported in Table 1, the Thr-Trp (TW) motif, which is within the T13 sequence, shows antioxidant activity, since it acts as a radical scavenger [45]. The presence of some antioxidant amino acid residues such as Tyr, Trp, Cys, and Met with electron/hydrogen-donating abilities is a determining factor for the radical scavenging activities of peptides. In general, it has been reported that Tyr- and Trp-containing dipeptides with Tyr/Trp residue at the N-terminus showed stronger antioxidant activities than at the C-terminus, and that the neighboring residue also affected their activities by steric effect, hydrophobicity, and hydrogen bonding, among others [45]. Based on this consideration and on the in silico prediction of the T13 secondary structure [11], it is reasonable to affirm that the TW motif of the BT13 may act a functional antioxidant tag (presumably because of its optimal exposure to an aqueous environment), making the BT13-based hydrogel intrinsically antioxidant. In addition, the contribution of biotin to the antioxidant activity of BT13 hydrogel might be considered irrelevant. Indeed, Turnaturi et al. clearly demonstrated that biotin is not effective in the in vitro scavenger activity of free radicals [46].

## 4. Conclusions

In summary, we reported the nanostructure and the hierarchical self-assembly propensity of a lupin-derived peptide belonging to the α-conglutin, through a biotin N-terminal tag.

By using CD spectroscopy, FT-IR spectroscopy, ThT-binding assay, and AFM analyses it was possible to establish that the biotin tag (1) favors the formation of ordered nanostructures with a typical β-sheet-rich amyloid-like nature, and (2) triggers a more oriented nanofiber distribution compared to the unbiotinylated peptide.

Furthermore, we obtained a thixotropic lupin-derived peptide hydrogel with tunable mechanical properties, becoming a promising candidate for future applications as an injectable scaffold for biomedicine, bioprinting, or drugs/cells delivery.

Lastly, we demonstrated for the first time that this naturally derived hydrogel has intrinsic antioxidant activity, useful for metabolic disorder or as a smart food additive. In addition, a preliminary screening of the lupin-derived peptide using BIOPEP suggested that the peptide may contain bioactive motifs such as inhibitors of ACE, alpha-glucosidase, and DPP-IV enzymes; these data will have to be better understood and further investigated in vitro in future studies in order to determine the potential bioactive multifunctional behavior of this peptide-based hydrogel.

The present work not only provides insights to fine tune nanostructures, mechanical properties, and antioxidant activities using a naturally occurring food peptide-based hydrogel, but also, combining food chemistry, nanotechnology, and synthetic biology approaches, it undoubtedly provides a proof-of-concept strategy to develop functional bio-inspired and more sustainable nano-nutraceuticals, starting from bioavailable β-sheet peptides obtained by enzymatic hydrolysis of the parent protein.

## Figures and Tables

**Table 1:** Screening of the T13 activity using BIOPEP database (www.uwm.edu.pI/biochemia). T13 Peptide sequence: LNALEPDNTVQSEAGTIETWNPK.

Activity	Sequence Motif
ACE inhibitor	AG
	GT
	IE
	LN
	EA
	ALEP
DPP-IV inhibitor	EP
	NP
	AL
	WN
	AG
	DN
	ET
	LN
	NA
	NT
	PK
	QS
	TW
	WQ
	TI
Antioxidant	TW
Alpha-glucosidase inhibitor	EA

ACE: angiotensin-converting enzyme; DPP-IV: dipeptidyl peptidase-IV.

## Data Availability

Data is contained within the article or supplementary material.

## Supplementary Materials

The following are available online at https://www.mdpi.com/2227-9059/9/3/294/s1, Figure S1: The α-conglutin protein sequence. Figure S2: Chemical structures of T13 and BT13 peptides. Figure S3: HPLC and MS spectra of T13 and BT13 peptides. Figure S4: Linear viscoelastic regime (LVR). Figure S5: Antioxidant activity of T13 peptide as plain solution.
