# Supplementary material for: Nanostructure, Self-Assembly, Mechanical Properties, and Antioxidant Activity of a Lupin-Derived Peptide Hydrogel
Source: Biomedicines. 2021 Mar 13;9(3):294. doi: 10.3390/biomedicines9030294 (PMC8000348; doi:10.3390/biomedicines9030294)
Supplement: Supplementary file 1 [file biomedicines-09-00294-s001.pdf]

biomedicines

Supplementary Materials

Nanostructure, Self-Assembly, Mechanical Properties, and Antioxidant Activity of a Lupin-Derived Peptide Hydrogel

```
10         20         30         40         50
MSNKLLALSL FLPFLLLFFC GCFASTFRQQ PQENECQFQR LNALEPDNTV
       60         70         80         90        100
QSEAGTIETW NPKNDELRCA GVALSRCTIQ RNGLRRPFYT NAPQEIYIQQ
      110        120        130        140        150
GRGIFGMIFP GCGETYEEPQ ESEKGQGPRP QDRHQKVEHF KEGDIIAVPT
      160        170        180        190        200
GIPFWMYNDG QTPVVAITLI DTTNLDNQLD QIPRRFYLSG NQEQEFLQYQ
      210        220        230        240        250
EKEGGQGQQQ EGGNVLSGFD DEFLEEALSV NKEIVRNIKG KNDDREGGIV
      260        270        280        290        300
EVKGGLKVII PPTMRPRHGR EEEEEEEEED ERRGDRRRRH PHHHHHEEEE
      310        320        330        340        350
EEEEEWSHQV RRVRRPHRHH HHRKDRNGLE ETLCTMKLRH NIGESTSPDA
      360        370        380        390        400
YNPQAGRFKT LTSIDFPILG WLGLAAEHGS IYKNALFVPY YNVNANSILY
      410        420        430        440        450
VLNGSAWFQV VDCSGNAVFN GELNEGQVLT IPQNYAAAIK SLSDNFRYVA
      460        470        480        490        500
FKTNDIPQIA TLAGANSEIS ALPLEVVAHA FNLNRDQARQ LKNNNPYKFL
      510
VPPPQSQLRA VA
```

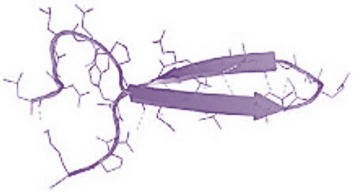

Figure 1. The α-conglutin protein sequence (11S globulin, legumin-like protein; Uniprot code Q53I54). In red the peptide sequence T13, used in this manuscript. The β-hairpin secondary structure of T13 peptides derived from parent α-conglutin protein sequence is shown below.

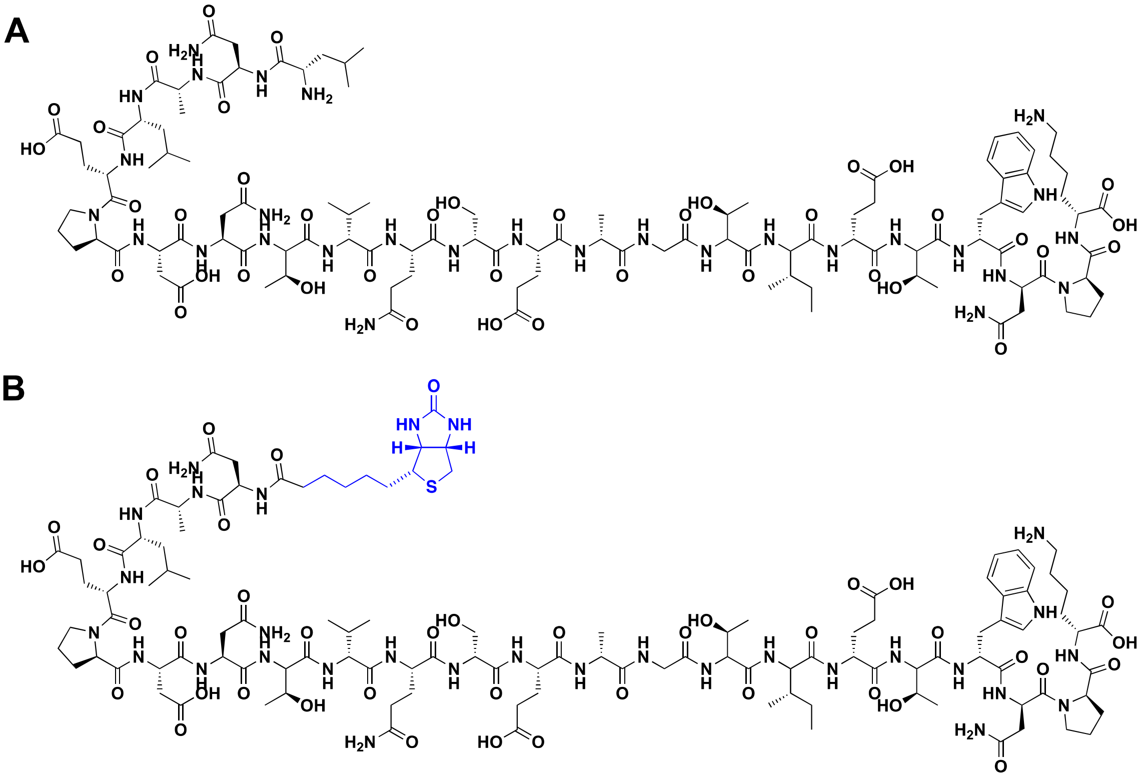

Figure 2. Chemical structures of (A) T13 and (B) BT13 peptides. In blue the linkage of biotinylation is shown.

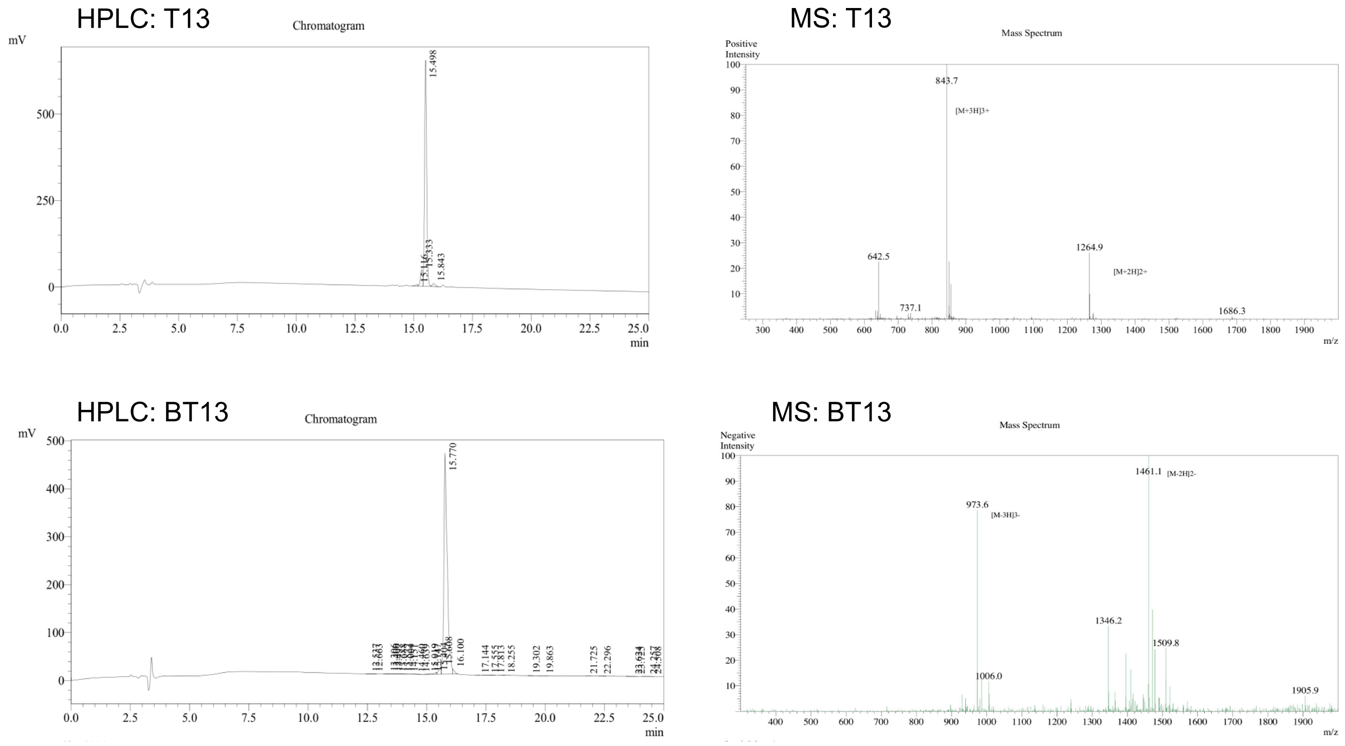

Figure 3. HPLC and MS spectra of T13 and BT13 peptides.

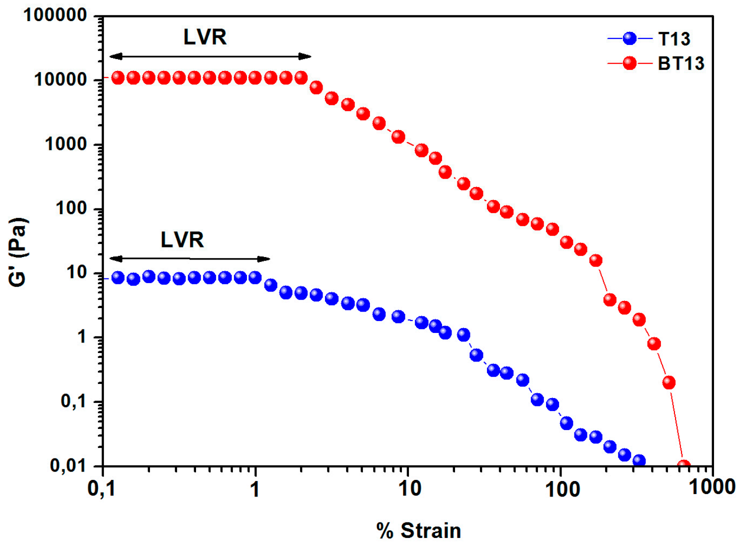

Figure 4. Linear viscoelastic regime (LVR). Strain sweep test to determine the LVR of T13 (in blue) and BT13 (in red) peptides.

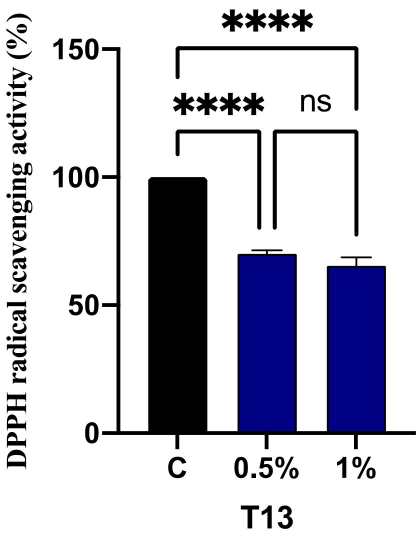

Figure 5. Antioxidant activity of T13 (in blue) as plain solution. The data points represent the means ± SD of three independent experiments in triplicate. Data were statistically analyzed by one-way ANOVA followed by Tukey's post-hoc test. (*) $p < 0.5$; (****) $p < 0.00001$. C: control sample.
